# A pre-hospital risk score predicts critical illness in non-trauma patients transported by ambulance to a Dutch tertiary referral hospital

**DOI:** 10.1186/s13049-021-00843-z

**Published:** 2021-02-12

**Authors:** Lars I. Veldhuis, Markus W. Hollmann, Fabian O. Kooij, Milan L. Ridderikhof

**Affiliations:** 1 Amsterdam UMC, Location AMC, Department of Emergency Medicine, Meibergdreef 9, Amsterdam, The Netherlands; 2 Amsterdam UMC, Location AMC, Department of Anesthesiology, Meibergdreef 9, Amsterdam, The Netherlands; 3 Amsterdam UMC, Location VUmc, Lifeliner 1 HEMS, De Boelelaan, 1117 Amsterdam, The Netherlands

**Keywords:** Clinical decision support systems, Critical illness, Emergency medical services, Pre-hospital care, Triage

## Abstract

**Background:**

Early pre-hospital identification of critically ill patients reduces morbidity and mortality. To identify critically ill non-traumatic and non-cardiac arrest patients, a pre-hospital risk stratification tool was previously developed in the United States. The aim of this study was to investigate the accuracy of this tool in a Dutch Emergency Department.

**Methods:**

This retrospective study included all patients of 18 years and older transported by ambulance to the Emergency Department of a tertiary referral hospital between January 1st 2017 and December 31st 2017. Documentation of pre-hospital vital parameters had to be available. The tool included a full set of vital parameters, which were categorized by predetermined thresholds.

Study outcome was the accuracy of the tool in predicting critical illness, defined as admittance to the Intensive Care Unit for delivery of vital organ support or death within 28 days. Accuracy of the risk stratification tool was measured with the Area Under the Receiver Operating Characteristics (AUROC) curve.

**Results:**

Nearly 3000 patients were included in the study, of whom 356 patients (12.2%) developed critical illness. We observed moderate discrimination of the pre-hospital risk score with an AUROC of 0.74 (95%-CI 0.71–0.77). Using a threshold of 3 to identify critical illness, we observed a sensitivity of 45.0% (95%-CI 44.8–45.2) and a specificity of 86.0% (95%-CI 85.9–86.0).

**Conclusion:**

These data show that this pre-hospital risk stratification tool is a moderately effective tool to predict which patients are likely to become critically ill in a Dutch non-trauma and non-cardiac arrest population.

## Introduction

### Background

While universally implemented tools exist for classifying the severity of trauma patients in the pre-hospital setting, early identification of non-trauma, potentially high-risk acute patient is much less uniform. Currently, triage of these patients is mainly based on the experience of ambulance personnel, distance to and facilities of the nearest hospital. Pre-hospital triage should be optimized, as early recognition of critically ill patients could lead to earlier use of lifesaving interventions and therefore potentially reduces morbidity and mortality [1]. Moreover, the treatment of critically ill patients should be regionalized, as previous studies have shown that Intensive Care Units (ICU) with higher volumes of high-risk patients have been associated with lower mortality rates [2–4]. As it is difficult to predict which patients are likely to deteriorate, and as identification of high-risk patients has proven to be challenging in the pre-hospital setting, several tools for predicting critical illness have been developed, such as the National Early Warning Score (NEWS) and the Modified Early Warning Score (MEWS) [5, 6]. The authors of a prospective study and the authors of a systematic review both concluded that the model developed by Seymour et al. is the best suitable model in predicting critical illness in out-of-hospital emergency care [7–9]. This risk stratification tool uses demographic and vital parameters to predict which patients are likely to become critically ill. According to this model, critically ill patients were older; had abnormal respiratory rates; lower pulse oximetry readings; lower systolic blood pressures; higher heart rates and lower Glasgow Coma Scales (GCS) compared to non-critically ill patients.

This risk stratification tool has been developed in a United States population including non-trauma and non-cardiac arrest patients who were transported to the Emergency Departments of both non-referral hospitals, as well as regional referral centers. Kievlan, et al. have validated the model for its use in a similar population in the United States [10]. This validation study showed an Area Under Receiver Operating Characteristics (AUROC) of 0.73.

However, accuracy and applicability of the risk stratification tool should be assessed for a European population.

## Materials and methods

### Aim of this investigation

The current study was undertaken to evaluate the use of a pre-hospital risk stratification tool in the Dutch health care system. The hypothesis was that the pre-hospital risk stratification tool is able to adequately discriminate between critically and non-critically ill patients, corresponding to an AUROC of at least 0.73.

### Study design, population and setting

Study approval was obtained by the Institutional Review Board prior to the start of the study (waiver W17_171 17.199). A formal ethical evaluation was not needed due to the retrospective nature of the study design. This was a retrospective cohort study in which all acute patients who presented to the Emergency Department of a Dutch tertiary referral hospital center were considered eligible for inclusion in the study in case they presented during the period between January 1st 2017 and December 31st 2017. Inclusion criteria were: age 18 years and older; transportation to the Emergency Department by ambulance and presence of pre-hospital documented vital parameters. Exclusion criteria were: patients with traumatic injuries; patients in cardiac arrest; interhospital transfers; those lacking more than four vital parameters needed to calculate the pre-hospital risk score and patients with a ‘do-not-resuscitate’ policy.

Dutch pre-hospital Emergency Medical Services (EMS) consist of a two-tiered system. The first-tier responders are ALS-trained ambulance nurses and drivers. The second-tier responders are physician-staffed teams called HEMS (Helicopter Emergency Medical Services), who are dispatched in a specific set of clinical situations; abnormal vital signs and upon specific request of an ambulance nurse. However, second-tier are usually dispatched mainly for pediatric resuscitations, severe traumatic injuries and/or need for (rapid sequence) endotracheal intubation.

### Data collection

As a standard procedure during daily clinical practice, the handover by ambulance personnel to the Emergency Department is sent in a digital format and added to the electronic hospital patient record. Data were extracted from this handover, as well as the hospital patient records. Extracted data included baseline parameters, such as age; sex and a full set of vital parameters (including respiratory rate, pulse oximetry readings, heart rate, blood pressure and GCS). In case more than one set of pre-hospital vital parameters were recorded, the first as well as the worst set of vital parameters were documented for analysis. Additionally, the following pre-hospital interventions were recorded as well: administration of oxygen (yes/no); including mode of delivery (nasal cannula, Non-Rebreathing Mask, Continuous Positive Airway Pressure-mask or endotracheal intubation) or the deployment of HEMS. In addition, we noted whether the patient lived in a nursing home or received specialized home care prior to the EMS encounter.

Information regarding ICU admittance and in-hospital mortality was obtained from the electronic hospital patient records. Patients were followed-up until discharge from the hospital and for at least 28 days after initial presentation at the Emergency Department.

### Outcomes

The primary outcome of the study was the accuracy of the risk stratification model in predicting critical illness, defined as admittance to the ICU anytime during hospitalization for delivery of vital organ support (mechanical ventilation or administration of vasopressors and / or inotropes) or death during admission to the hospital or after discharge. This included all causes of mortality occurring in the hospital as well as after discharge during the first 28 days after initial presentation at the Emergency Department.

According to the risk stratification model by Seymour, et al. age, pulse oximetry, respiratory rate, systolic blood pressure, heart rate and GCS score were categorized to predetermined thresholds and summed to calculate the total pre-hospital risk score (range 0–8) for each encounter (Table 1) [8].

**Table 1:** Variables according to the risk stratification model by Seymour, et al

Model variable	
	Point scores
Age, years	
< 45	0
45 to 64	1
≥ 65	1
Systolic blood pressure, mmHg	
≤ 90	1
91 to 140	0
141 to 180	0
> 180	0
Heart rate, beats per minute	
≤ 60	0
61 to 99	0
100 to 119	0
≥ 120	1
Respiratory rate, breaths per minute	
< 12	1
12 to 23	0
24 to 35	1
≥ 36	2
Oxygen saturation, %	
≥ 93	0
88 to 92	0
80 to 87	1
< 80	1
Glasgow Coma Scale score	
15	0
12 to 14	1
8 to 11	1
< 8	2

### Sample size

The sample size was calculated with a two-sided 95%-Confidence Interval (95%-CI) for a single proportion. According to a prior study the observed proportion of critical illness was 0.05 [8]. Utilizing these numbers with a *p*-value of 0.01, this resulted in a sample size of 1825 patients. It was estimated that recruiting Emergency Department patients during a year would yield sufficient patients.

### Model assessment and data analysis

Recorded data were presented as absolute values with percentages and continuous data as mean values with standard deviations or median values with interquartile ranges, depending on whether data were normally distributed. Normal distribution of variables was assessed with Kolmogorov-Smirnoff tests, as well as exploring frequency distributions (histograms). Numerical variables with a normal distribution were evaluated using the Students *t*-test or the Mann-Whitney U test in case there was no normal distribution. Chi-square analysis was used for statistical testing of categorical data. To reduce bias, patients in whom more than four out of six variables were missing required to calculate individual risk scores, were excluded from the study. In at least one and less than five variables missing, single imputation with normal value substitution for vital parameters that were not measured, was utilized.

Regarding the primary outcome, the hypothesis was that the risk stratification tool could distinguish between patients who would become critically ill or not. This was tested by means of assessing model accuracy using a specified reference value (i.e. AUROC curve) with binominal 95%-CIs. For this statistical analysis the total pre-hospital risk score was the independent variable. In addition to the AUROC, sensitivity and specificity were calculated for each risk score in the range from 0 to 8. A cut-off point of four points as optimum threshold was used, equal to the original model [8].

As a sensitivity analysis, the AUROC curve using the worst pre-hospital vital signs rather than the initial vital signs were utilized, as this would reflect patient deterioration more accurately. To determine if our handling of missing data introduced bias, an additional analysis was performed with only those patients with a full set of vital parameters. Lastly, the AUROC curve was calculated in case critical illness was defined as admittance to the ICU or death within 72 h. All analyses were performed with SPSS (version 23.0; SPSS, Inc., Chicago, IL). All tests of significance used a two-sided *p* < 0.05.

## Results

**Fig. 1:**
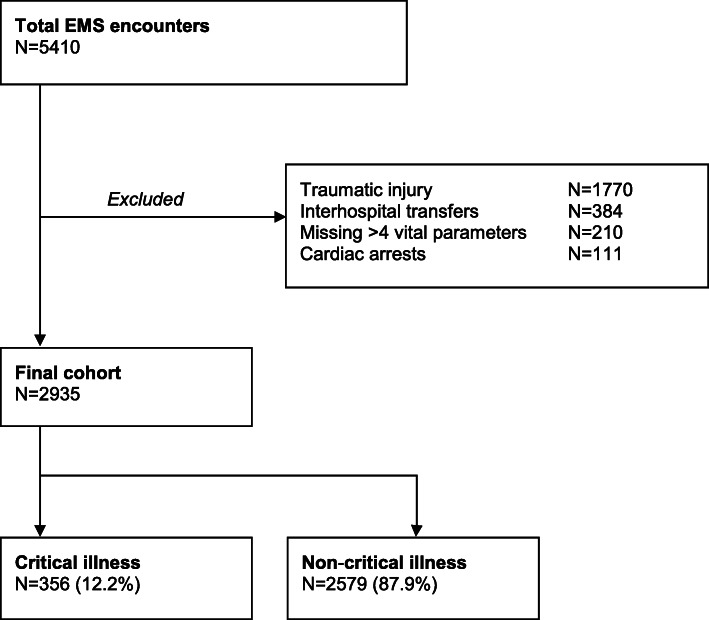
EMS encounter selection, exclusion and included patients. *Abbreviations:* EMS Emergency Medical Services

During the study period, a total of 5410 patients were transported to the Emergency Department by ambulance. Of these patients, 2935 were included in the study (Fig. 1). Reasons for exclusion were: traumatic injury (*n* = 1770); inter-hospital transfers (*n* = 384); missing more than four vital parameters (*n* = 210); and cardiac arrest (*n* = 111).

Critical illness occurred in 356 patients (12.2%). Compared to encounters in which patients did not develop critical illness, critically ill patients were older, had pre-hospital respiratory symptoms more frequently and had higher heart rates and lower GCS scores (*p* < 0.01 for all), as can be seen in Table 2. However, systolic blood pressure was not significantly different between groups (*p =* 0.29).

**Table 2:** Patients demographics and clinical characteristics

	Critical Illness	Non-Critical Illness	*P* Value
Number of patients (%)	356 (12.1)	2579 (87.9)	
Number of patients with imputed data (%)	52 (14.6)	414 (16.1)	
Age, years, median (IQR)	67 (56–77)	61 (47–74)	< 0.01
Male sex, n (%)	200 (56)	1349 (52)	0.08
Initial pre-hospital vital signs, median (IQR)			
Oxygen Saturation, %	95 (88–98)	97 (95–98)	< 0.01
Respiratory rate breaths/minute	20 (14–27)	16 (14–20)	< 0.01
Systolic blood pressure, mmHg	140 (113–168)	140 (121–160)	0.29
Heart rate, beats/minute	93 (74–111)	86 (73–102)	< 0.01
Glasgow Coma Scale score	15 (10–15)	15 (15–15)	< 0.01
Diagnostic category by ambulance personnel, n (%)			
Neurological	128 (36)	740 (29)	
Internal	111 (31)	881 (34)	
Pulmonology	55 (15)	267 (10)	
Cardiovascular	25 (7)	345 (13)	
Surgical	11 (3)	96 (4)	
Urological	6 (2)	50 (2)	
Psychiatric/toxicologic	3 (< 1)	73 (3)	
Medical (NOS)	17 (5)	127 (5)	
Pre-hospital interventions, n (%)			
Airway - noninvasive	157 (44)	446 (17)	< 0.01
Airway - endotracheal intubation	5 (1)	0 (0)	< 0.01
Physician staffed team	22 (6)	0 (0)	< 0.01
General practitioner at scene, n (%)	61 (18)	404 (16)	0.26
Nursing Home before encounter, n (%)	51 (15)	264 (10)	0.01

*Abbreviations*: *IQR* Interquartile Range, *NOS* not otherwise specified

As expected, critically ill patients received pre-hospital airway maneuvers more often than patients in the control group. The involvement of general practitioners did not significantly differ between the groups. However, the prevalence of living in a nursing home prior to the EMS encounter (*p* = 0.01), and the number of patient encounters assisted by HEMS was significiantly higher for critically ill patients (*p* < 0.01). The amount of patients in each subgroup of critical illness is listed in Table 3.

**Table 3:** Components of critical illness

**Total number of critically ill patients - n (%)**	356 (12.1)
**Mortality < 28 days without ICU admittance- n (%)**	97 (27)
**ICU admittance - n (%)**	259 (73)
**Need for mechanical ventilation**	127 (36)
**Hemodynamic instability**	40 (11)
**Combination respiratory insufficiency and hemodynamic instability**	92 (26)
**Died**	65 (25)

*Abbreviations*: *ICU* Intensive Care Unit

In total, 198 (8%) non-critically ill, and eight (2%) critically ill patients were transported to a different hospital.

### Primary outcome

After calculating the total pre-hospital risk score ranging from 0 to 8 for each encounter, the AUROC curve was calculated for developing critical illness. The pre-hospital risk stratification tool had an AUROC of 0.74 (95%-CI 0.71–0.77).

Based on the AUROC, sensitivity and specificity were calculated for each individual pre-hospital risk score and are shown in Table 4. When the prior set optimum threshold score of four was utilized, a sensitivity of 24.6% (95%-CI 24.4–24.8) and a specificity of 95.4% (95%-CI 95.3–95.4) were observed in identifying critical illness. However, with a threshold score of three, the sensitivity increased to 45% (95%-CI 44.8–45.2) and the specificity decreased to 86% (95%-CI 85.9–86.0).

**Table 4:** Sensitivity and specificity per pre-hospital score

Pre-hospital risk cut off points	0	1	2	3	4	5	6	7	8
**Sensitivity,**	99.2	98.3	75.8	45.0	24.6	9.2	2.1	0.4	0.2
**(95%-CI)**	(99.2–99.3)	(98.3–98.4)	(75.7–76.0)	(44.8–45.2)	(24.4–24.8)	(9.1–9.3)	(2.0–2.1)	(0.4–0.4)	(0.2–0.2)
**Specificity,**	4.1	12.2	62.9	86.0	95.4	98.4	99.8	100	100
**(95%-CI)**	(4.1–4.1)	(12.1–12.2)	(62.3–63.0)	(85.9–86.0)	(95.3–95.4)	(98.3–98.4)	(99.8–99.8)	(99.9–100)	(100–100)

*Abbreviations*: 95%-CI 95%-Confidence Interval

Using the worst set of pre-hospital vital parameters instead of the primary vital parameters resulted in an AUROC of 0.74 (95%-CI 0.71–0.77). This did not improve model performance significantly, compared to the original model (*p* = 0.98), as can be seen in Table 5. Additionally, in case critical illness was defined as death or ICU admittance within 72 h, the AUROC was 0.72 (95%-CI 0.69–0.77). The model performance did not significantly improve (*p* = 0.56).

**Table 5:** Discrimination of the pre-hospital risk score in the primary model and sensitivity analyses

	AUROC	95%-CI
**Original model**	0.74	0.71–0.77
**Worst set of vital parameters**	0.74	0.71–0.77
**Patients without missing data**	0.75	0.72–0.78
**ICU admittance or death < 72 h after presentation**	0.72	0.69–0.77

## Discussion

The results of the current study show that a pre-hospital risk stratification tool had moderate discrimination for predicting critical illness in adult non-trauma and non-cardiac arrest patients presented to the Emergency Department of a Dutch tertiary referral hospital.

In the original study by Seymour, et al.*,* using the pre-determined cut-off point of four resulted in a sensitivity of 22% and a specificity of 98% in predicting occurrence of critical illness. Our data show a similar accuracy with a sensitivity of 25% and a specificity of 95%.

Using the cut-off point of four, the risk stratification tool had a very high specificity but the sensitivity was moderate to low. Therefore the chances of over-triage are low, however, critically ill patients with a low score might not be recognized by utilizing this tool, which leads to an increased rate of false negative results.

However, using a cut-off score of three in our study, the sensitivity increased to 45% and the specificity decreased to 86%, improving the clinical use of this tool. Using the worst set of pre-hospital vital parameters or defining critical illness as admittance to the ICU or death within 72 h, did not improve the performance of the risk stratification tool in a sensitivity analysis. With a high specificity, patients with a score of at least three are likely to become critically ill. However, as sensitivity is relatively low, there is a relatively high risk in missing critically ill patients.

Knowing the high specificity and thereby the high chances of becoming critically ill, this risk model may assist the EMS in deciding whether the patient should be treated in a referral hospital rather than a non-referral hospital. However, prospective multicenter studies are needed to confirm our findings. In addition, a wearable device constantly measuring all the vital parameters may assist in recognizing deteriorating patients [11]. The model by Seymour, et al. is not only superior to similar risk stratification models for its model accuracy, but due its applicability in the pre-hospital setting, earlier recognition and pre-alerting of patients that are at high risk of becoming critically ill, can be achieved. This has the potential to reduce morbidity as well as mortality.

Critical illness was defined as admittance to the ICU or death during the first 28 days after presentation. The original model also included severe sepsis in their definition of critical illness [8].

In the current study, there was a relatively high frequency of critically ill patients of 12%. In previous studies this was 4.5% [8, 10]. These studies included not only multiple referral centers of expertise, but also non-referral hospitals, possibly treating patients who were less ill.

Critically ill patients received supplemental oxygen more often compared to non-critically ill patients. While the risk stratification tool consists of pulse oximetry and respiratory rate, it does not consider the delivery of supplemental oxygen. However, the use of supplemental oxygen has a direct impact on pulse oximetry and occasionally indirectly on respiratory rate [12]. Recently, the National Early Warning Score (NEWS) had been adjusted with implementing supplemental oxygen to the score model, resulting in the NEWS2. However, these risk models are only focusing on mortality rather than ICU admittance [13, 14]. In addition, a multicenter study showed no benefits of NEWS 2 compared to NEWS [15].

### Limitations of the study

This was a chart review study with its inherent bias due to its retrospective design.

Patients who did not have a set of pre-hospital vital signs were excluded from this study and selection bias was introduced, as “scoop-and-run” patients often have no full set of vital parameters measured pre-hospitally, but are obviously critically ill. This selection bias was reduced as much as possible by only excluding those patients with missing more than four out of six required parameters. Additionally, data imputation was performed in case of missing data. Nevertheless, it must be emphasized that as missing data were imputed as normal values, the amount of disturbed vital parameters might have been underestimated. Sensitivity analysis including only patients without missing values, showed no significant difference in predicting critical illness compared to the patients that had missing data imputed. But again, as missing data were imputed as normal values this potentially resulted in concerning bias, as abnormal values may have been underestimated.

The use of beta-blockers was not documented in the current study. These medications have impact on heart rate as well as blood pressure and occurrence of sinus tachycardia is potentially masked. Therefore, use of these drugs have the potential to have biased the study results. It was not possible to show the impact of these medications on the accuracy of the model. Further research should elucidate this.

To prevent any misclassification of patients becoming critically ill or not, it was verified whether the included patients died within 28 days after initial presentation or were admitted to the ICU. However, it was not possible to ascertain outcomes in patients who were initially admitted to the tertiary referral hospital, in which the study was performed and who were transferred to another hospital during the study period. Even so, none of the patients were transferred to different hospitals without being seen by an emergency physician, and in addition, it is unlikely that unstable patients were transported to non-referral hospitals.

## Conclusions

Use of this pre-hospital risk stratification tool moderately discriminates between potential critically ill and non-critically ill adult patients in the acute setting and may assist in recognizing critically ill patients in the pre-hospital setting.
